# Spectrum of genomic variations in Indian patients with progressive familial intrahepatic cholestasis

**DOI:** 10.1186/s12876-018-0835-6

**Published:** 2018-07-04

**Authors:** Anjali Sharma, Ujjal Poddar, Shikha Agnihotry, Shubha R. Phadke, Surender K. Yachha, Rakesh Aggarwal

**Affiliations:** 1 Department of Gastroenterology, Sanjay Gandhi Postgraduate Institute of Medical Sciences, Lucknow, 226014 India; 2 Department of Pediatric Gastroenterology, Sanjay Gandhi Postgraduate Institute of Medical Sciences, Lucknow, 226014 India; 3 Department of Medical Genetics, Sanjay Gandhi Postgraduate Institute of Medical Sciences, Lucknow, 226014 India

**Keywords:** Progressive familial intrahepatic cholestasis, Hereditary cholestasis, Genetic variation, Polymorphism

## Abstract

**Background:**

Progressive familial intrahepatic cholestasis (PFIC) is caused by variations in ATP8B1, ABCB11 or ABCB4 genes. Data on genetic variations in Indian patients with PFIC are lacking.

**Methods:**

Coding and splice regions of the three genes were sequenced in unrelated Indian children with PFIC phenotype. The variations identified were looked for in parents, 30 healthy persons and several variation databases, and their effect was assessed in-silico.

**Results:**

Among 25 children (aged 1–144 months), nine (36%) had unique major genomic variations (ATP8B1: 4, ABCB11: 3 and ABCB4: 2). Seven had homozygous variations, which were assessed as ‘pathogenic’ or ‘likely pathogenic’. These included: (i) four amino acid substitutions (ATP8B1: c.1660G > A/p.Asp554Asn and c.2941G > A/p.Glu981Lys; ABCB11: c.548 T > C/p.Met183Thr; ABCB4: c.431G > A/p.Arg144Gln); (ii) one 3-nucleotide deletion causing an amino acid deletion (ATP8B1: c.1587_1589delCTT/p.Phe529del); (iii) one single-nucleotide deletion leading to frame-shift and premature termination (ABCB11: c.1360delG/p.Val454Ter); and (iv) a complex inversion of 4 nucleotides with a single-nucleotide insertion leading to frame-shift and premature termination (ATP8B1: c.[589_592inv;592_593insA]/p.Gly197LeufsTer10). Two variations were found in heterozygous form: (i) a splice-site variation likely to cause abnormal splicing (ABCB11: c.784 + 1G > C), and (ii) a nucleotide substitution that created a premature stop codon (ABCB4: c.475C > T/p.Arg159Ter); these were considered as variations of uncertain significance. Three of the nine variations were novel.

**Conclusions:**

Nine major genomic variations, including three novel ones, were identified in nearly one-third  of Indian children with PFIC. No variation was identified in nearly two-thirds of patients, who may have been related to variations in promoter or intronic regions of the three PFIC genes, or in other bile-salt transport genes.

**Electronic supplementary material:**

The online version of this article (10.1186/s12876-018-0835-6) contains supplementary material, which is available to authorized users.

## Background

Progressive familial intrahepatic cholestasis (PFIC) is a group of heterogeneous liver disorders of childhood with disruption of normal secretion of bile salts and/or phospholipids into the bile [1]. It accounts for nearly 10 to 15% of cases with cholestasis in early childhood, and for a large proportion of pediatric liver transplantation procedures [1]. PFIC is classified into three types, namely PFIC1, PFIC2 and PFIC3, based on detection of causative variations in ATP8B1, ABCB11 and ABCB4 genes, respectively [1]. Several different variations have been reported in each of these genes [2–4]. In PFIC1 and PFIC2, symptoms appear during infancy, whereas in PFIC3, these may be delayed to late childhood or adolescence. Also, characteristically, the serum levels of gamma-glutamyl transpeptidase (GGT) are normal in PFIC1 and PFIC2, but are elevated in PFIC3 [5]. Since the protein encoded by ATP8B1 is also expressed in other body tissues, some PFIC1 patients also have extrahepatic manifestations, such as diarrhea, sensorineural deafness, pancreatic deficiency and growth retardation [1, 6]. All three forms of PFIC are inherited in autosomal recessive manner [1].

The genomic variations underlying PFIC show geographic heterogeneity. Thus, some variations, such as G308V, D554N and I661T in ATP8B1 gene have been observed frequently in Caucasian groups [2, 7], but not in other populations; in these latter groups, other sequence variations have been identified as causing PFIC [8]. Further, in a substantial number of patients with PFIC phenotype, no variation in these three genes can be identified. Some of these cases are related to variations in other genes involved in the secretion of bile salts [9].

Data on PFIC in the Indian population are limited to a few case reports and case series [10–15]. In particular, no information is available on genetic variations in Indian patients with PFIC, except in one patient that we reported recently [16]. Given the genetic heterogeneity underlying the three forms of PFIC, particularly between populations [17], the genomic variations in Indian patients with PFIC may be expected to differ from those reported elsewhere. We therefore decided to study genomic variations in the exonic and splice site regions of the ATP8B1, ABCB11 and ABCB4 genes in a group of Indian children with clinical features suggestive of PFIC.

## Methods

### Study patients

The study included children with liver disease with clinical and biochemical findings that suggested a diagnosis of PFIC. The diagnosis was considered in all infants and children presenting with cholestatic liver disease, with no evidence of biliary obstruction on imaging or of another cause of liver injury. Cholestasis was defined in children as jaundice with itching and deranged liver function tests, and in neonates as jaundice with conjugated bilirubin exceeding 20% of total serum bilirubin (or > 1.0 mg/dL when total bilirubin was < 5.0 mg/dL). All children underwent clinical and laboratory work-up, including screening for TORCH (toxoplasmosis, rubella, cytomegalovirus and herpes virus) group of neonatal infections among infants, screening for metabolic liver disease (non-glucose reducing substances in the urine and assay for  galactose-1-phosphate uridyl transferase in red blood cells; serum alpha-fetoprotein levels; and, serum ferritin level and minor salivary gland biopsy in those suspected to have neonatal hemochromatosis), and Alagille syndrome (slit lamp examination, X-ray spine and echocardiography). For patients with symptoms starting after 3 years of age, biochemical tests and liver biopsy examination were done to exclude Wilson disease and autoimmune hepatitis.

Biochemical liver function tests including serum gamma-glutamyl transpeptidase (GGT) levels were measured in all patients. Those with elevated GGT levels were considered to have PFIC3, and those with normal GGT were considered to have PFIC1 or PFIC2. All patients with PFIC3 underwent MRCP; however, this was done selectively in those with PFIC1/PFIC2. All patients also underwent a liver biopsy as part of their diagnostic workup (not for this study); however, immunohistochemistry for bile salt export protein (BSEP; to diagnose PFIC2) and for multidrug resistance 3 protein (MDR3; to diagnose PFIC3) were not possible.

In families with multiple cases, only one child was studied. For each child, clinical and biochemical data were recorded; in addition, family history of liver disease and history of prominent itching during third trimester of pregnancy in the mother were also noted, and a pedigree tree was drawn with special emphasis on consanguinity. From each subject, venous blood (~ 2 ml) was collected in EDTA, after obtaining written consent from one of the parents, and genomic DNA was extracted using the phenol-chloroform method [18]. Blood specimens were also collected from their parents, wherever possible. The study was approved by our institution’s Ethics Committee.

### Laboratory methods and data analysis

From the genomic DNA, all the coding exons (exons 2 to 28 for each gene) and the surrounding splice site regions were amplified using polymerase chain reaction with specific primers for genes ATP8B1 [19], ABCB11 [20] and ABCB4 [21]. The PCR products were subjected to Sanger sequencing in both directions using a 3130 Genetic Analyzer (Applied Biosystems/ThermoFisher).

The nucleotide sequencing data obtained were analysed using Finch TV (1.4.0) (http://jblseqdat.bioc.cam.ac.uk/gnmweb/download/soft/FinchTV_1.4/doc/) and Seqtrace (version 0.9.0) (https://code.google.com/archive/p/seqtrace/) softwares. The sequences were then aligned against genomic and messenger RNA reference sequences for ATP8B1 (NG_007148.2, NM_005603.4), ABCB11 (NG_007374.1, NM_003742.2) and ABCB4 (NG_007118.1, NM_000443.3) genes from National Centre for Biotechnology Information (NCBI) database. Each variation observed was manually verified.

The sequence variation identified were looked up in various genomic variation databases [NCBI dbSNP (https://www.ncbi.nlm.nih.gov/projects/SNP/), Human Gene Mutation Database (HGMD) (http://www.hgmd.cf.ac.uk/ac/index.php), Exome Aggregation Consortium (ExAC) (http://exac.broadinstitute.org/), ClinVar NCBI (https://www.ncbi.nlm.nih.gov/clinvar/), 1000 genome browsers (http://www.internationalgenome.org/1000-genomes-browsers/)]. Further, the effect of each variation on the amino acid sequence of the respective protein was assessed; this was followed by prediction of the effect of amino acid change on the protein function using several bioinformatics tools, namely PROVEAN [22], Mutation Taster [23], Polyphen 2 [24], PhD-SNP [25], SIFT [26], SNAP [27], Meta SNP [28]. The effect of sequence variations located near splice sites was predicted using Human Splicing Finder tool [29].

Each genomic sequence variant was then analysed for its pathogenic significance using the standards and guidelines laid down by the American College of Medical Genetics and Genomics and the Association for Molecular Pathology [30]. In brief, these guidelines recommend that sequence variants in a gene should be classified into five categories -- ‘pathogenic’, ‘likely pathogenic’, ‘uncertain significance’, ‘likely benign’, and ‘benign’ -- based on frequency of the variant in the healthy population, computational data, functional data and segregation data.

### Studies in parents and control subjects

Any ‘pathogenic’ or ‘likely pathogenic’ variations identified in the patients were looked for in the parents by amplification and sequencing of the specific exons of the affected gene.

For non-synonymous variations identified in one or more patients, the frequency of minor alleles was assessed by sequencing the genomic DNA from 30 healthy persons.

## Results

The 25 patients included in this study were unrelated to each other, and were aged 1 month to 12 years (median: 15 months; 19 boys). Their clinical and laboratory findings are shown in Table 1. Seven patients were born of consanguineous marriages.

**Table 1:** Clinical and laboratory features of patients included in the study (*n* = 25)

Clinical feature	Value in all patients studied (*n* = 25)	Value in those with genomic variation (*n* = 9)	Value in those without genomic variation (*n* = 16)	*p* value (with genomic variation versus without genomic variation)
Age at presentation (months)	15 (1–144)	15 (3–144)	23 (1–48)	0.089
Age at onset of symptoms (months)	5 (0.25–138)	3.5 (0.25–138)	5 (0–18)	1.123
Male (number)	19 (76%)	7	12	1.000
History of consanguinity (number)	7 (28%)	5	2	0.058
History of cholestasis of pregnancy (number)	10 (40%)	3	7	0.691
Intense pruritus (number)	24 (96%)	8	16	0.360
History of skin bleeding (number)	17 (68%)	5	12	0.394
Jaundice (number)	25 (1)	9	16	1.000
Weight z score^a^	−2.08 (−3.55 to +0.32)	− 2.35 (−3.55 to +0.32)	−1.98 (−3.52 to +0.21)	0.205
Height z score^b^	− 1.63 (−5.62 to +2.26)	−1.55 (−4.75 to +0.2)	−1.73 (−5.62 to +2.26)	0.478
Palpable liver below right costal margin (cm)^c^	4 (1–8)	3 (2–8)	4 (1–7)	0.790
Palpable spleen below left costal margin (cm)^d^	2 (1–5)	1 (1–5)	3 (2–4)	0.300
Total serum bilirubin (mg/dL)	7.0 (1.3–32.0)	12.7 (1.3–21.0)	6.6 (1.7–32.0)	0.296
Serum albumin (g/dL)^e^	3.7 (2.7–4.5)	3.8 (3.2–4.2)	3.8 (2.7–4.5)	0.777
Serum aspartate aminotransferase (U/L)	175 (45–1210)	92 (45–1120)	214 (69–1210)	0.844
Serum alanine aminotransferase (U/L)	142 (29–682)	96 (29–598)	104 (30–682)	0.947
Serum gamma-glutamyl transpeptidase (U/L)	28 (8–69)	25 (8–57)	33.5 (11–69)	0.154

Data are shown as median (range), or as number (%)

^a^Values less than 2.0 were observed in 13 children, including 5/9 with and 8/16 without genomic variations (*p* = 1.00)

^b^Values less than 2.0 were observed in 10 children, including 4/9 with and 6/16 without genomic variations (*p* = 1.00)

^c^Liver was palpable in all the 25 children

^d^Spleen was palpable in 16 of the 25 children: including 5/9 with and 11/16 without genomic variations (*p* = 0.67)

^e^Serum albumin level was abnormally low (<3.5 g/dL)  in 3 of 25 children: including 1/9 with and 2/16 without genomic variations (*p* = 1.00)

### Pathogenic or likely pathogenic sequence variations

**Fig. 1:**
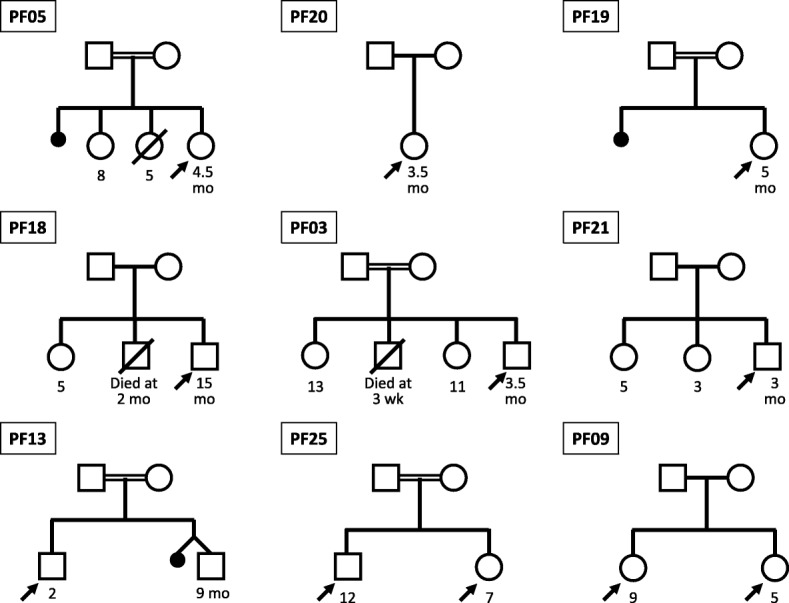
Family tree for 9 patients in whom major variations in ATP8B1 (patients PF05, PF20, PF19 and PF18), ABCB11 (patients PF03, PF21 and PF13) or ABCB4 (patients PF25 and PF09) genes were found. Five of these nine  patients had history of consanguinity. Clinical, biochemical and histological findings in these patients are included in Table 3

Overall, 9 patients were found to have 9 different major genomic variations (Table 2). Of these, seven were present in homozygous form and each of these was interpreted either as ‘pathogenic’ or ‘likely pathogenic’ (*n* = 7). The remaining two variations were present in heterozygous form and were assessed to be ‘of uncertain significance’ (*n* = 2); in both these patients, no other pathogenic variation was identified. Five of the 7 patients with homozygous variations and none of the two with heterozygous variations reported consanguinity (Fig. 1**)**.

**Table 2:** ‘Pathogenic’ or ‘Likely pathogenic’ genomic variations identified in Indian patients with PFIC syndrome

Gene showing sequence variation	Subject ID	History of consanguinity	Nature of variation	Nucleotide change	Type of mutation	AA change	In silico prediction	Inclusion in databases^a^	Population frequency (in ExAC)
‘Pathogenic’/ likely pathogenic variations									
ATP8B1	PF05	Yes	Homozygous	c.[589_592inv;592_593insA] GGAG ⇒ CTCCA	Frame-shift; truncation after 205 amino acids	p.Gly197LeufsTer10	Deleterious	Novel^b^	
	PF20	No	Homozygous	c.1587_1589delCTT CTT ⇒ -	In-frame deletion of one amino acid	p.Phe529del	Deleterious	rs756395915, 18:55351308 CAAG/C	8.24 × 10^− 6^
	PF19	Yes	Homozygous	c.1660G > A	Mis-sense (GAT⇒AAT)	p.Asp554Asn	Deleterious	rs121909101, CM004386 ClinVar:7269	
	PF18	No	Homozygous	c.2941G > A	Mis-sense (GAG⇒AAG)	p.Glu981Lys	Deleterious	CM096608	
ABCB11	PF03	Yes	Homozygous	c.548 T > C	Mis-sense (ATG ⇒ ACG)	p.Met183Thr	Deleterious	CM103530	
	PF13	Yes	Homozygous	c.1360delG	Truncation after 453 amino acids	p.Val454*	Deleterious	Novel	
ABCB4	PF25	Yes	Homozygous	c.431G > A	Mis-sense (CGA ⇒ CAA)	p.Arg144Gln	Likely deleterious	rs863225299, ClinVar:217883	3.3 × 10^− 5^
Variation of ‘uncertain significance’									
ABCB11	PF21	No	Heterozygous^c^	c.784 + 1 G > C	Splice site variation	Possible abnormal splicing	Deleterious	Novel	
ABCB4	PF09	No	Heterozygous	c.475C > T	Truncation after 474 amino acids (CGA ⇒ TGA)	p.Arg159*	Deleterious	rs377160065, CM075939, 7:87082321 G/A	3.2 × 10^− 5^

^a^This column shows identification details of the particular sequence variant in large-scale human mutation databases, i.e. dbSNP, ExAC, HGMD and ClinVar

^b^This variation found in our cohort has also been reported as a single case report previously [16]

^c^This patient also had homozygous p.Val444Ala variation in ABCB11 gene

None of the nine variations shown in this table was found in 1000 genome database

Of the nine patients with significant variations, four had these variations in the ATP8B1 gene, 3 in ABCB11 gene and 2 in ABCB4 gene. Of the nine variations identified, three (ATP8B1: 1, ABCB11: 2) were novel. Four variations led to a single amino acid substitution, one led to shifting of reading frame and premature truncation, two led to creation of a stop codon at the variation site, one led to an in-frame deletion of one amino acid, and one was predicted to lead to loss of a splice site. The clinical and laboratory findings in the patients with identifiable variations are shown in Table 3. A comparison of these findings with those in patients without such variations (Table 1**)** showed no significant difference between the two groups, except that patients with genomic variations tended to be younger and more often had history of consanguinity, compared to those without variations.

**Table 3:** Clinical features of patients in whom pathogenic or likely pathogenic genomic variations were identified

Gene showing sequence variation	Subject ID	AA change	Age at onset (mo)	Clinical features	Extra-hepatic manifestations	Cholestasis of pregnancy in mother	Liver biopsy	Serum bilirubin (total/conjugated) (mg/dl)	ALT^a^ (IU/L)	AST^b^ (IU/L)	GGT^c^ (IU/L)	SAP^d^ (IU/L)	Presence of identical variation in parents	
													Father	Mother
ATP8B1	PF05	p.Gly197LeufsTer10	3.5	Jaundice, intense pruritus (4 mo), skin bleeds	Stunting	Yes	Intracytoplasmic and intracanalicular cholestasis, and paucity of interlobular bile ducts	7.1/5.0	29	45	8	630	Heterozygous	Heterozygous
	PF20	p.Phe529del	0.25	Jaundice (0.25 mo), pruritus (4 mo), skin bleeds	Stunting	–	Intracytoplasmic and intracanalicular cholestasis, and paucity of bile ducts	15.0/11.0	115	92	12	661	Not available for testing	Heterozygous
	PF19	p.Asp554Asn	3	Jaundice, pruritus (4.5 mo)	–	–	Bland cholestasis and paucity of bile ducts	21.0/8.0	59	220	43	883	Not available for testing	Heterozygous
	PF18	p.Glu981Lys	3	Jaundice, intense pruritus (6 mo), skin bleeds	–	–	Intracytoplasmic and intracanalicular cholestasis, focal bile duct proliferation	19.4/13.9	35	75	9	856	Not available for testing	Heterozygous
ABCB11	PF03	p.Met183Thr	5	Intermittent jaundice, intense pruritus, skin bleeds	Steatorrhea, wheeze, stunting	–	Unremarkable portal tracts	1.3/0.6	45	62	29	393	Heterozygous	Heterozygous
	PF21	Possible abnormal splicing	1	Jaundice (1 mo), pruritus (6 mo)	–	Yes	Distorted architecture, giant cells, cholestasis, and paucity of bile ducts	9.6/5.8	598	918	57	1374	Variation absent	Heterozygous
	PF13	p.Val454*	6	Jaundice, pruritus, ascites (21 mo)	–	–	Cholestasis, giant cells, bile duct proliferation, and bridging fibrosis	12.7/7.2	264	226	16	337	Heterozygous	Not available for testing
ABCB4	PF25	p.Arg144Gln	138	Jaundice	Stunting	Yes	Distorted architecture, bridging fibrosis, and cholestasis	15.0/7.7	279	1120	356	461	Heterozygous	Heterozygous
	PF09	p.Arg159*	54	Jaundice, pruritus, skin bleeds	–	–	Maintained architecture, mild portal fibrosis, no bile duct proliferation	3.6/2.2	96	84	25	919	Heterozygous	Variation absent

^a^*ALT* alanine aminotransferase (reference value: up to 40 IU/L), ^b^*AST* aspartate aminotransferase (reference value: up to 40 IU/L), ^c^*GGT* gamma glutamyl transpeptidase (reference value: up to 86 IU/L), ^d^*SAP* serum alkaline phosphatase (reference value: up to 150 IU/L for adults, up to 450 IU/L for children)

The patient in this table had a median (range) age of 15 (3–144) months, and included 7 boys and 2 girls

The results of bioinformatic prediction of the effect of these genomic variations are shown in Additional file 1: Table S1. None of the nine variations included in Table 2 was observed in any of the 30 healthy controls. Among patients with homozygous variations (*n* = 7), both parents were available for testing for four patients and one parent each was available for three patients; all of them showed the respective variation in heterozygous form. For the two patients with heterozygous variations, one parent each was available for testing; in either case, the tested parent too had the variation in a heterozygous form.

### Non-pathogenic variations

Three non-synonymous variations were identified in several children with PFIC (Table 4). These variations also had a high frequency in various population databases as well as in our patients as well as controls, indicating their benign nature. Further, all the three non-synonymous variations were predicted by various bioinformatics software tools to be neutral (Additional file 1: Table S2). Hence, these were considered as non-pathogenic.

**Table 4:** ‘Benign’ or ‘likely benign’ non-synonymous genomic variations identified in Indian patients with PFIC syndrome

Gene	Nucleotide change	AA change	Inclusion in databases^a^			Allele frequency reported in ExAC	Allele frequency reported in 1000 genome browser	Allele frequency observed in 25 patients in the current study	Allele frequency observed in 30 controls in the current study
			dbSNP ID	ExAC ID	HGMD				
ABCB11	c.1331 T > C	p.Val444Ala	rs2287622	2:169830328 A/G	CM071525	0.5794	0.5887	0.580 (Homozygous: 9 Heterozygous: 11)	0.667 (Homozygous: 13 Heterozygous 14)
	c.1772A > G	p.Asn591Ser	rs11568367	2:169826592 T/C	CM044555	0.0158	0.0310	0.080 (Homozygous: 1 Heterozygous: 2)	0.100 (Homozygous: 0 Heterozygous: 6)
ABCB4	c.1954A > G	p.Arg652Gly	rs2230028	7:87056176 T/C	CM072814	0.1056	0.1703	0.140 (Homozygous: 1 Heterozygous: 5)	0.133 (Homozygous: 1 Heterozygous: 6)

All these variations were predicted to be benign by all the five bioinformatics tools used (Provean, PhD-SNP, SIFT, SNAP, Meta SNP)

^a^This column shows identification details of the particular sequence variant in large-scale human mutation databases, i.e. dbSNP, ExAC, HGMD and ClinVar

In addition, 12 synonymous variations (Additional file 1: Table S3) were also identified in several patients. These too had a high frequency in population databases as well as in our patient population, and hence were considered as non-pathogenic.

## Discussion

In our study of 25 unrelated Indian children with PFIC phenotype, we found pathogenic or likely-pathogenic sequence variations in ATP8B1, ABCB11 or ABCB4 gene in seven and variations of unknown significance in two. In these 9 patients, a total of nine different variations, including three novel variations, were identified. The variations which are particularly frequent in the Caucasian and other populations were not found. No major variation was found in the remaining 16 kindreds.

No data are available on the genomic variations responsible for PFIC from India. The Indian population is genetically diverse with contribution from at least two highly divergent populations – i.e. the 'North Indians’, genetically close to the Middle Easterners, Central Asians, and Europeans, and a distinct group termed as ‘Ancestral South Indians’ [31–33], with some contributions from the sub-Saharan and Middle Eastern lineages [34]. This may explain the diversity in genetic variations observed in our cohort, identification of novel variations and the difference in variations in our patients from those reported in the other population groups. The diversity of variations in our patients suggests that it will be difficult to identify a panel of few common variations for diagnostic testing in Indian patients with PFIC.

We encountered three novel variations. One of these (p.Gly197LeufsTer10) was expected to lead to production of a truncated ATP8B1 protein (of 205-amino acid length instead of the usual 1251 amino acids), which would lack a part of the E1-E2 ATPase domain (aa 170–234), and the entire cation ATPase (aa 533–628) and phospholipase ATPase C domains (aa 919–1174), and hence be non-functional. This patient has been described in detail elsewhere as a case report [16]. Another variation (p.Val454*) led to a truncated ABCB11 protein. Both these truncation variations would be clearly expected to be pathogenic even without any functional studies to study their biological effect. The third novel variation affected a splice donor site in the ABCB11 gene, and was heterozygous. Demonstration of its functional effect needed sequencing of the patient’s liver transcriptome; however, a liver biopsy for this purpose was considered ethically unacceptable. This patient also had a heterozygous p.Val444Ala variation, which is known to cause a reduction in enzyme activity, and this may explain the occurrence of disease in him despite the novel splice site variation being heterozygous.

Variations observed in our remaining six patients had been reported previously in other population groups. Of these, three were in ATP8B1 gene and had been previously shown to be associated with disease. First, an in-frame deletion (p.Phe529del) had been previously reported in two patients – in homozygous form in a Japanese patient with severe disease needing liver transplant by 4 years of age [2, 35], and in compound heterozygous form with a splice site variation in a patient of mixed Caucasian-African American ancestry [2]. Our patient was homozygous for this variation and had a severe illness. Second, a p.Asp554Asn substitution had been previously reported in three Inuit patients [19, 36, 37]. It affects a centrally-located aspartate residue in the AASPDEGALV motif, specific for P-type ATPases [38], and was associated in an in vitro study with an absent expression of ATP8B1 on canalicular membranes [39]. Third, a p.Glu981Lys substitution had been previously reported in a Japanese patient with PFIC [40], and the mutant protein was shown to have a reduced interaction with its substrate leading to a nearly 80% reduction in its activity in a yeast-cell model [41].

Of the remaining three previously-reported variations, one was in ABCB11 gene. This variation in ABCB11 gene (p.Met183Thr) had been previously reported in a patient with PFIC2, who had a compound heterozygous state [42]; in our patient, this variation was homozygous. Of the two previously-known variations in the ABCB4 gene identified in our patients, one (p.Arg159*) was previously reported in an Italian patient in heterozygous state [43], with no genomic variation on the other allele. This is similar to the findings in our patient, who had an elder sibling with similar illness, and in whom the symptoms appeared at 54 months of age. Given that PFIC3 is well recognized to have an autosomal recessive inheritance, one would expect another variation on the other allele of this gene, which however could not be identified. The other ABCB4 variation (p.Arg144Gln) that we observed has not been reported in patients with PFIC; however, it is likely to be pathogenic since it was predicted to be deleterious, was present in homozygous form and had a low population frequency.

We also identified some missense and synonymous variations which were frequent in population-level genetic databases, and were predicted not to adversely affect protein structure or function, and hence were considered ‘benign’ or ‘likely benign’. One of these, the c.1331 T > C (p.Val444Ala) substitution in ABCB11 gene may need particular mention. This variation has previously been variably reported to be associated with reduced activity of the protein and disease [44]. As discussed above, this substitution was identified in a patient who also had a heterozygous novel splice-site variation in this gene, and hence could have played in role in disease causation in that patient.

Importantly, no coding region or splice-site variation was identified in any of the three genes studied in 16 of our 25 patients. This could have several explanations. In studies from other geographical regions too, variations are identified in only 30–50% of cases with PFIC phenotype [2, 42]. Second, we did not look for variations in the promoter and non-coding regions; however, such variations are only infrequently responsible for disease. Third, our methods could miss a heterozygous major deletion; however, such a deletion would need a pathogenic variation on the other allele to cause disease. Fourth, we did not study variations in other genes involved in bile acid transport (such as MYO5B, TJP2, NR1H4, FXR or VIPAS39) [45–49]. It should be possible to work around some of these limitations in future, by using whole exome or whole genome sequencing. Finally, it is possible that some of our patients may not have had PFIC, even though we took precautions to exclude other liver diseases.

As expected, we identified genomic variations more often in patients with history of consanguinity (5/7; 71%) than in those without (4/18; 22%). In the two patients who had homozygous variations in the absence of history of consanguinity, the parents may have had some shared ancestry which was missed on history taking; this is possible with close inbreeding prevalent in some communities in India.

## Conclusions

Our study identified major genomic variations in only about one-third of Indian children with PFIC. None of the nine variations that we identified  was particularly frequent, and three of these were novel. Further, our findings suggest that genomic variations responsible for PFIC in Indian patients may differ from those in other populations. Since we did not find any genomic variation in nearly two-thirds of our patients, it may be useful to undertake whole-exome or whole-genome sequencing studies in Indian children with PFIC to look for variations in promoter/intronic regions of the ATP8B1, ABCB11 and ABCB4 genes and in other genes involved in bile salt transport.

## Additional file

Additional file 1: **Table S1.** Results of prediction of effect, using various bioinformatic tools, of various ‘pathogenic’ or ‘likely pathogenic’ non-synonymous genomic variations. **Table S2.** Results of prediction of effect, using various bioinformatic tools, of various ‘neutral’ non-synonymous genomic variations. **Table S3.** Synonymous genomic variations identified in Indian patients with PFIC syndrome (DOCX 24 kb).

## Abbreviations

BSEP: Bile salt export pump
ExAC: Exome Aggregation Consortium
GGT: Gamma-glutamyl transpeptidase
HGMD: Human Genome Mutation Database
MDR3: Multidrug resistance protein 3
NCBI: National Centre for Biotechnology Information
PFIC: Progressive familial intrahepatic cholestasis
