# Supplementary material for: Spectrum of genomic variations in Indian patients with progressive familial intrahepatic cholestasis
Source: BMC Gastroenterol. 2018 Jul 4;18:107. doi: 10.1186/s12876-018-0835-6 (PMC6032793; doi:10.1186/s12876-018-0835-6)
Supplement: Supplementary file 1 — Table S1. Results of prediction of effect, using various bioinformatic tools, of various ‘pathogenic’ or ‘likely pathogenic’ non-synonymous genomic variations. Table S2. Results of prediction of effect, using various bioinformatic tools, of various ‘neutral’ non-synonymous genomic variations. Table S3. Synonymous genomic variations identified in Indian patients with PFIC syndrome (DOCX 24 kb). [file 12876_2018_835_MOESM1_ESM.docx]

**Table S1: Results of prediction of effect, using various bioinformatic tools, of various ‘pathogenic’ or ‘likely pathogenic’ non-synonymous genomic variations.**

| **Gene** | **Amino acid change** | **Bioinformatic tool** | | | | | | | | | | | | | | |
| --- | --- | --- | --- | --- | --- | --- | --- | --- | --- | --- | --- | --- | --- | --- | --- | --- |
|  |  | **PROVEAN** | | **PhD-SNP** | | **SIFT** | | **SNAP** | | **Meta-SNP** | | **Mutation Taster**  **(Amino acid conservation scores)** | | | **Polyphen2** | |
|  |  | **Score** | **Predicted effect** | **Score** | **Predicted effect** | **Score** | **Predicted effect** | **Score** | **Predicted effect** | **Score** | **Predicted effect** | **PhyloP** | **Phast-cons** | **Predicted effect** | **Score** | **Predicted effect** |
| ATP8B1 | F529del | −7.074 | D | Not applicable | | Not applicable | | Not applicable | | Not applicable | | 0.20 | 0.95 | D | Not applicable | Not applicable |
|  | D554N | −4.811 | D | 0.831 | D | 0 | D | 0.79 | D | 0.921 | D | 5.99 | 0.99 | D | 1.000 | D |
|  | E981K | −3.146 | D | 0.662 | D | 0.01 | D | 0.71 | D | 0.729 | D | 5.06 | 1.00 | D | 0.999 | D |
| ABCB11 | M183T | −3.809 | D | 0.769 | D | 0 | D | 0.605 | D | 0.759 | D | 4.86 | 1.00 | D | 1.000 | D |
| ABCB4 | R144Q | −3.148 | D | 0.777 | D | 0.02 | D | 0.635 | D | 0.653 | D | 1.91 | 1.00 | D | 0.997 | D |

PROVEAN: Variants with a score equal to or below −2.50 are considered ‘deleterious’

PhD-SNP: Scores range between 0 and 1.0; variations with values >0.50 are predicted as disease-causing

SIFT: Values range between 0 and 1.0; variations with values >0.05 are predicted as neutral

SNAP: Output normalized between 0 and 1; variations with values >0.50 are predicted as deleterious

Meta-SNP: Values range between 0 and 1.0; variations with values >0.50 are predicted as disease-causing

PhyloP: Values vary between -14 and +6, the closer the value is to +6, the more probable that the nucleotide is conserved

Phastcons: Values vary between 0 and 1, the closer the value is to 1, the more probable that the nucleotide is conserved

Polyphen2: Score ranges between 0 and 1.0; values 0.0 to 0.15 predicted as benign, 0.15 to 1.0 as possibly deleterious, and with 0.85 to 1.0 as deleterious

D: Deleterious

**Table S2: Results of prediction of effect, using various bioinformatic tools, of various ‘neutral’ non-synonymous genomic variations.**

| **Gene** | **Amino acid change** | **Bioinformatic tool** | | | | | | | | | | | | | | |
| --- | --- | --- | --- | --- | --- | --- | --- | --- | --- | --- | --- | --- | --- | --- | --- | --- |
|  |  | **PROVEAN** | | **PhD-SNP** | | **SIFT** | | **SNAP** | | **Meta-SNP** | | **Mutation Taster**  **(Amino acid conservation scores)** | | | **Polyphen2** | |
|  |  | **Score** | **Predicted effect** | **Score** | **Predicted effect** | **Score** | **Predicted effect** | **Score** | **Predicted effect** | **Score** | **Predicted effect** | **PhyloP** | **Phast-cons** | **Predicted effect** | **Score** | **Predicted effect** |
| ABCB11 | V444A | 0.007 | Neutral | 0.286 | Neutral | 0.71 | Neutral | 0.4 | Neutral | 0.413 | Neutral | -0.17 | 0.90 | Neutral | 0.001 | Neutral |
|  | N591S | −0.841 | Neutral | 0.144 | Neutral | 0.71 | Neutral | 0.315 | Neutral | 0.212 | Neutral | 2.17 | 1.00 | Neutral | 0.98 | Deleterious |
| ABCB4 | R652G | −0.048 | Neutral | 0.389 | Neutral | 0.39 | Neutral | 0.605 | Neutral | 0.447 | Neutral | -0.29 | 0 | Neutral | 0.00 | Neutral |

PROVEAN: Variants with a score equal to or below −2.50 are considered ‘deleterious’

PhD-SNP: Scores range between 0 and 1.0; variations with values >0.50 are predicted as disease-causing

SIFT: Values range between 0 and 1.0; variations with values >0.05 are predicted as neutral

SNAP: Output normalized between 0 and 1; variations with values >0.50 are predicted as deleterious

Meta-SNP: Values range between 0 and 1.0; variations with values >0.50 are predicted as disease-causing

PhyloP: Values vary between -14 and +6, the closer the value is to +6, the more probable that the nucleotide is conserved

Phastcons: Values vary between 0 and 1, the closer the value is to 1, the more probable that the nucleotide is conserved

Polyphen2: Score ranges between 0 and 1.0; values 0.0 to 0.15 predicted as benign, 0.15 to 1.0 as possibly deleterious, and with 0.85 to 1.0 as deleterious

**Table S3: Synonymous genomic variations identified in Indian patients with PFIC syndrome**

| **Gene** | **Nucleotide change** | **Amino acid location** | **Inclusion in databases*** | | | **Allele frequency reported in ExAC** | **Allele frequency in 1000 genome database** | **Number with this variant sequence among 25 patients** | **Allele frequency observed in patients** | **Number with this variation among 30 controls** | **Allele frequency observed in controls** |
| --- | --- | --- | --- | --- | --- | --- | --- | --- | --- | --- | --- |
|  |  |  | **dbSNP** | **ExAC** | **HGMD** |  |  | **Homozygous / Heterozygous / None** |  | **Homozygous/ Heterozygous/ None** |  |
| ATP8B1 | c.696T>C | p.Asp232= | rs319438 | 18:55364852 A/G |  | 0.99800 | 0.9946 | 25 / 0 / 0 | 1.00 | Not available |  |
|  | c.811A>C | p.Arg271= | rs319443 | 18:55362532 T/G |  | 0.99720 | 0.9926 | 25 / 0 / 0 | 1.00 | Not available |  |
| ABCB11 | c.108T>C | p.Asp36= | rs3815675 | 2:169870855 A/G |  | 0.03037 | 0.0631 | 0 / 2 / 23 | 0.04 | Not available |  |
|  | c.807T>C | p.Tyr269= | rs2287616 | 2:169847412 A/G |  | 0.03064 | 0.0647 | 0 / 2 / 23 | 0.04 | Not available |  |
|  | c.957A>G | p.Gly319= | rs7563233 | 2:169842746 T/C | CM092797 | 0.04403 | 0.1218 | 0 / 1 / 24 | 0.02 | Not available |  |
|  | c.1971G>T | p.Val597= | rs11568371 | 2:169826573 C/A |  | 0.01593 | 0.0312 | 1 / 2 / 22 | 0.08 | 0 / 0 / 30 | 0.00000 |
|  | c.3084A>G | p.Ala1028= | rs497692 | 2:169789016 T/C | CM092757 | 0.55570 | 0.4968 | 18 / 3 / 4 | 0.78 | Not available |  |
| ABCB4 | c.175C>T | p.Leu59= | rs2302387 | 7:87092185 G/A |  | 0.17520 | 0.2524 | 0 / 4 / 21 | 0.08 | Not available |  |
|  | c.504C>T | p.Asn168= | rs1202283 | 7:87082292 G/A | CM096001 | 0.47650 | 0.3478 | 3 / 9 / 13 | 0.30 | 6 / 12 / 12 | 0.40000 |
|  | c.525G>C | p.Thr175= | rs558416191 | 7:87082271 C/T |  | 0.00002 | 0** | 0 / 1 / 24 | 0.02 | 0 / 0 / 30 | 0.00000 |
|  | c.711A>T | p.Ile237= | rs2109505 | 7:87079406 T/A | CM031116 | 0.21010 | 0.2614 | 1 / 8 / 16 | 0.20 | Not available |  |
|  | c.1938T>C | p.Asp646= | rs553616378 | 7:87056192 A/G |  | 0.00328 | 0.0044 | 0 / 1 / 24 | 0.02 | 0 / 2 / 28 | 0.03333 |

* This column shows identification details of the particular sequence variant in large-scale human mutation databases, i.e. dbSNP, ExAC and HGMD

** This variation was not found but G>C variation at this locus was found in 1 allele (allele frequency = 0.0005).
